# A comparison of individual force decline profiles during a fatiguing eccentric trunk flexion and extension protocol: a pilot study

**DOI:** 10.3389/fspor.2024.1431607

**Published:** 2024-08-21

**Authors:** Yasemin Paksoy, David Kpobi, Jakob Henschke, Lucie Risch, Tilman Engel

**Affiliations:** University Outpatient Clinic, Sports Medicine and Sports Orthopedics, University of Potsdam, Potsdam, Germany

**Keywords:** muscle fatigue, torque output, eccentric exercise, variability, trunk muscles

## Abstract

**Introduction:**

Muscle fatigue, characterized by diminished force production and contraction sustainability, can impair muscle coordination and increase joint instability. Differing force profiles used in fatiguing tasks, such as prolonged eccentric trunk protocols, might provide insights into individualized strategies and resulting spinal stability. Thus, this study assessed individual differences in fatigue characteristics during an eccentric trunk flexion-extension protocol in a population of asymptomatic individuals.

**Methods:**

Twelve participants (2 f/10 m, 29 ± 4 years, 78.4 ± 16.9 kg, 1.76 ± 0.10 m) performed an eccentric trunk flexion and extension protocol on an isokinetic dynamometer (45° flexion to 10° extension; 60°/s), with final analysis on 8 participants for trunk flexion and 11 for trunk extension due to data exclusions. Participants engaged in a maximal all-out (AO) task for 2 min. Each participant's torque output (Nm) was assessed on a repetition-by-repetition basis, and smoothened by a moving average of 5 repetitions. Individual time profiles for reaching fatigue thresholds (10%, 15%, 20% and 30% reduction of initial torque output), and inter subject variability (by coefficient of variation, CV in %) were assessed throughout the AO task. Further, percentage torque reduction and variability were assessed at mid (1-minute) and end (2-minute) of task.

**Results:**

On average, for flexor and extensor muscles combined, participants reached a force reduction of 10% within 23.2 ± 19.1 s, of 15% within 44.9 ± 19.6 s, of 20% in 62.4 ± 26.3 s, and of 30% within 79.2 ± 21.8 s. The variability between individuals for the timepoint of reaching the defined torque thresholds was assessed by CV ranged between 23.4% and 103.8% for trunk flexor muscles, and between 28.4% and 56.5% for trunk extensor muscles.

**Discussion:**

A reduction of up to 20% was seen on average for all participants within 1-minute of eccentric trunk flexion and extension. Different inter-individual force output profiles were seen throughout the AO protocol, potentially related to physiological, skill-based, technical, adaptational, and/or motivational factors. The increase in fatigue resulted in a reduction in variability among individuals. A 2-minute protocol effectively induced pronounced fatigue, offering insights into individual force profiles and strategies.

## 1 Introduction

Muscle fatigue, a non-specific symptom that affects the performance and function of the musculoskeletal system during sportive and daily life activities (1), can be defined as the transient inability of a muscle to maintain a given level of force or power output (2). Muscle fatigue reduces the capacity of the muscle to generate and sustain force, alters the coordination and control of movement, impairs the reaction time and shock absorption ability, and increases the muscle weakness and susceptibility to injury (3–5). Therefore, identifying the timepoint and degree of muscle fatigue in individuals can prevent unnecessary strains in the muscles, and ultimately injuries and musculoskeletal problems (1, 6).

Changes in the recruitment pattern of trunk muscles due to muscle fatigue have also been posited to have postural implications that challenge the stability of the spine (7, 8). Various studies demonstrated that the occurrence of trunk muscle fatigue reduces muscle coordination, increases spinal instability, and even lead to impairments in postural control (9–11). Johanson et al. (2011) have suggested that acute muscle fatigue in back muscles can act as a precipitating factor for the onset or exacerbation of low-back pain (11). Lin et al. (2009) have reported that, compared to ankle and knee muscles, muscle fatigue in the lower back has the most significant effect on postural control (12). However, since the complex and multi-joint structure of the trunk makes isolated assessments difficult, it has been researched only scarcely (13).

Over the past years, research has documented how muscular fatigue occurs during various types of tasks and attempted to clarify the underlying mechanisms (14). Baroni et al. (2011) have suggested that maximal isokinetic eccentric contractions, due to their higher torque production, provide a more effective way of assessing fatigue through increased mechanical overload (15). Alternatively, Lou et al. (2012) assessed muscle fatigue during maximal isometric contractions, finding considerable force declines within a period of less than a minute (16). Regardless of the assessment method, a precise starting point for the onset of fatigue is still debated in literature, due to the gradual nature of muscle fatigue (3). Still, a decrease in torque of about 10%–15% is commonly defined as threshold for the onset of muscle fatigue (17).

Due to interindividual differences in neuromuscular adaptations, varying strategies to maintain torque output in the presence of fatigue might be deployed (18, 19). This can be attributed to differences in their capacity to recruit additional muscle fibers or motor units, with some individuals relying more on metabolic processes and alternative compensatory mechanisms (19, 20). These individual differences in coping strategies may lead to different patterns of force decline, in response to an identical fatiguing protocol.

The majority of available studies compared force or power outputs at the start and end of the testing protocol exclusively. However, it has not been reported so far, at which time point muscle fatigue is reached (according to predefined threshold of force reduction) and how torque profiles vary between individuals. Therefore, the aim of the present pilot study was to assess fatigue characteristics during an eccentric trunk flexion-extension protocol and to identify differences and the time required to reach different fatigue thresholds in a population of asymptomatic individuals. We hypothesized that participants would exhibit individual differences in the time required to reach predefined fatigue thresholds during the protocol, reflecting varying neuromuscular adaptations and compensatory strategies to maintain torque output.

## 2 Materials and method

### 2.1 Participants

A convenience sample was recruited by announcements at the University campus and the surrounding local area. All participants met the following criteria: age between 20 and 55 years, being pain-free/asymptomatic prior to the measurement day, no previous history of back pain in the last six months, and being capable of English literacy. Exclusion criteria were any apparent musculoskeletal, vascular or neurological injury, surgery or illness within the last six months, acute infection/cold, or severe and debilitating pain that contraindicates physical activity (21, 22). All participants provided written informed consent prior to enrollment. The study was conducted in accordance with the ethical standards for scientific research, including adherence to the principles outlined in the Helsinki Declaration, and was supervised by the medical board of the University Outpatient Clinic. Ethical approval was given by the local university ethics committee (registration number: 26/2022).

### 2.2 Study design and procedure

This investigation employed an experimental pilot study design. A clinical anamnesis and examination by a physician were conducted prior to the measurement to clarify the eligibility for participation in the study. Additionally anthropometric data were documented in a standardized case report form. Training load, defined as the weekly hours of training, was also collected for each participant. Prior to the fatigue protocol a concentric warm-up trial (30 repetitions of concentric trunk flexion-extension at approx. 50%–70% of the subjectively predicted maximum force) and an eccentric familiarization trial (5 repetitions of eccentric trunk flexion-extension at approx. 50% of the subjectively predicted maximum force) were performed, followed by a resting period of 5 min. The subsequent testing protocol comprised two parts: (1) 5 repetitions maximal eccentric trunk flexion-extension contraction (MVC5) followed by 3 min of rest, and (2) a 2 min all-out (AO) maximal eccentric trunk flexion-extension fatiguing protocol. Participants also provided subjective ratings of perceived exertion (RPE) using the Borg Scale (ranging from 6 to 20, where 6 indicates no exertion at all and 20 indicates maximal exertion) before and after the fatigue protocol (23).

An isokinetic dynamometer (Con-trex dynamometer MJ with adapter type TP-1000, Physiomed, Germany) was used to measure torque output (Nm) during trunk flexion and extension movements. Participants were placed inside the dynamometer with an upright standing position and slight knee flexion (10° flexion). To maintain the position, pads were placed on the knee, waist and chest. The test was performed in a movement range between 10° of trunk extension and 45° trunk flexion, at a rotational velocity of 60°/s. During the eccentric task individuals were told to maximally resist the movement of the machine, at every repetition.

All conducted measurements were performed at the biomechanics laboratory of the university outpatient clinic at a constant time of the day (midday).

### 2.3 Data processing and analysis

Maximum torque (mean value of the three highest out of five repetitions) of the MVC5 task was used as reference torque output for the AO task. To account for random fluctuations of torque output, a moving average filter over 5-repetitions was employed to the data recordings of the AO task (24). Subsequently, peak torque output of each participant was expressed as percentage value of the MVC5 reference recording. Fatigue thresholds were defined by reduction of the participant´s initial torque output of 10%, 15%, 20%, and 30% during AO task based on common thresholds as provided by literature (17, 25). Important notice: always the first time point when the individual reached the respective threshold (based on the smoothed signal by the moving average technique) was assessed for later outcome presentation.

Demographic characteristics were presented descriptively as mean ± standard deviation (SD). Variability between individuals for timepoint of reaching the defined torque thresholds during AO was assessed by analysis of coefficient of variation (CV; standard deviation divided by the mean, and expressed as percentage value) for each threshold (10%, 15%, 20%, 30%) and each muscle group (trunk flexors, trunk extensors). Distribution of the individual's torque output during AO tasks was displayed using boxplot graphs and via individual line graphs plotted over the trial time.

## 3 Results

In total, 12 healthy adults (10 males and 2 females; age 29 ± 4 years; height 1.76 ± 0.10 m; weight 78.4 ± 16.9 kg; body mass index 25 ± 4; 5.7 ± 5.1 training hours per week), participated in the study. Due to implausible data or initial torque output below 90% of the MVC5 task, the torque outputs measured during trunk flexion in four participants and trunk extension in one participant data were excluded from the data analysis. Consequently, the analysis focused on the torque output data from 8 participants during trunk flexion and 11 participants during trunk extension. The detailed descriptive characteristics of the study group are shown in Table 1.

**Table 1:** Descriptive statistics of study population (mean ± SD).

Sex (*N*)	Female (2)	Male (11)
Age (yr)	27 ± 7	29 ± 4
Height (m)	1.59 ± 0.06	1.78 ± 0.07
Weight (kg)	52.5 ± 0.7	80.7 ± 15.6
BMI	21 ± 2	25 ± 4
RPE Pre-AO	8.0 ± 2.8	8.8 ± 2.7
RPE Post-AO	16.5 ± 0.7	17.3 ± 2.8

Yr, years; m, meter; kg, kilogram; BMI, body mass index; SD, standard deviation.

The MVC5 values for trunk flexor and trunk extensor muscles are presented in Table 2. For all participants, the MVC5 value for trunk flexor and extensor muscles combined was found 283.5 ± 123.5 Nm, with a range of 82.2–522.3 Nm.

**Table 2:** MVC5 values separated for trunk flexor and trunk extensor muscles (descriptive data with mean ± SD, range).

Muscle group	MVC5 mean ± SD (Nm)	MVC5 range (min-max) (Nm)
Trunk flexor muscles	333.9 ± 113.6	144.3–522.3
Trunk extensor muscles	233.1 ± 115.7	82.2–488.9

SD, standard deviation; Min, minimum; Max, maximum; Nm, Newtonmeter.

On average, for flexor and extensor muscles combined, participants reached a force reduction of 10% within 23.2 ± 19.1 s, of 15% within 44.9 ± 19.6 s, of 20% within 62.4 ± 26.3 s, and of 30% within 79.2 ± 21.8 s. The coefficient variation (CV) ranged between 23.4% and 103.8%, depending on the muscle group and fatigue thresholds. Results of the % decline, and CV analysis for trunk flexor muscles and trunk extensor muscles are summarized in Table 3.

**Table 3:** Time points, range and variability of the different fatigue thresholds during AO task, separated for trunk flexor and trunk extensor muscles (descriptive data with mean ± SD, range and CV).

		Mean ± SD (s)	Range (min-max) (s)	Coefficient variation (%)
Trunk flexor muscles	10% decrease	21.6 ± 22.4	2.4–74.4	103.8
	15% decrease	44.9 ± 21.6	19.2–81.6	48.1
	20% decrease	60.9 ± 25.2	31.2–100.8	41.4
	30% decrease	71.7 ± 16.8	55.2–108.0	23.4
Trunk extensor muscles	10% decrease	25.5 ± 14.4	4.8–40.8	56.5
	15% decrease	45.3 ± 18.5	7.2–67.2	40.9
	20% decrease	64.5 ± 29.4	9.6–98.4	45.6
	30% decrease	88.0 ± 25.0	50.4–120.0	28.4

SD, standard deviation; Min, minimum; Max, maximum; s, seconds.

**Figure 1:**
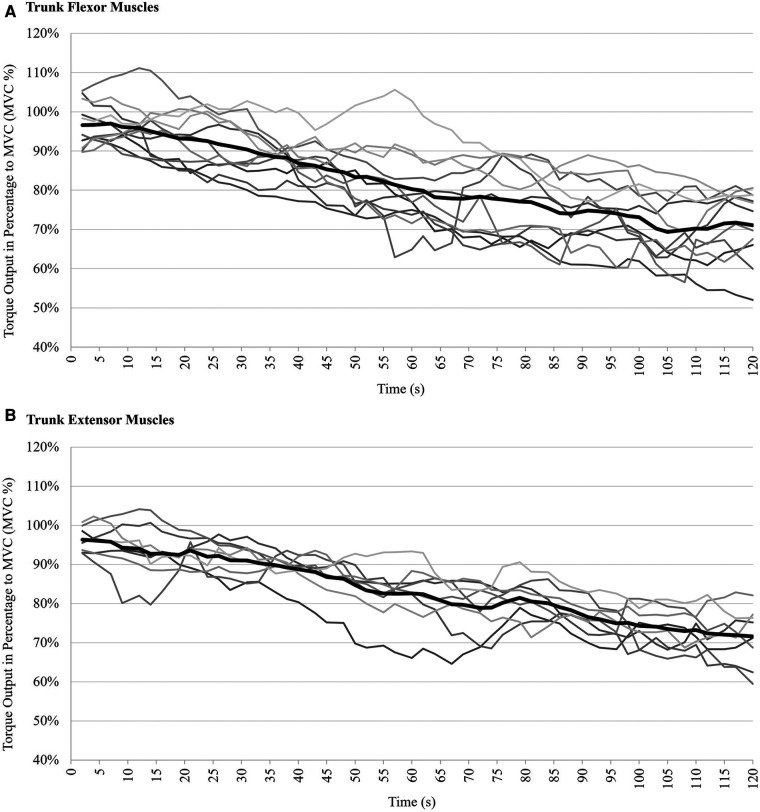
Torque output of trunk muscles during 2-minute AO task, expressed as a percentage of their maximum voluntary contraction during MVC5: **(A)** trunk flexor muscles (during eccentric trunk extension), **(B)** trunk extensor muscles (during eccentric trunk flexion). Light grey lines represent the torque output of each participant, solid black lines represent the mean torque output during eccentric trunk flexion and extension.

Individual courses of trunk flexor and extensor torque output during the AO task for each participant and averaged across all participants are shown in Figure 1. Furthermore, each participant's individual torque output plotted over time is provided in Supplementary File S1.

Subjective ratings of perceived exertion were 8.6 ± 2.6 Pre-AO and 16.3 ± 3.6 Post-AO.

**Figure 2:**
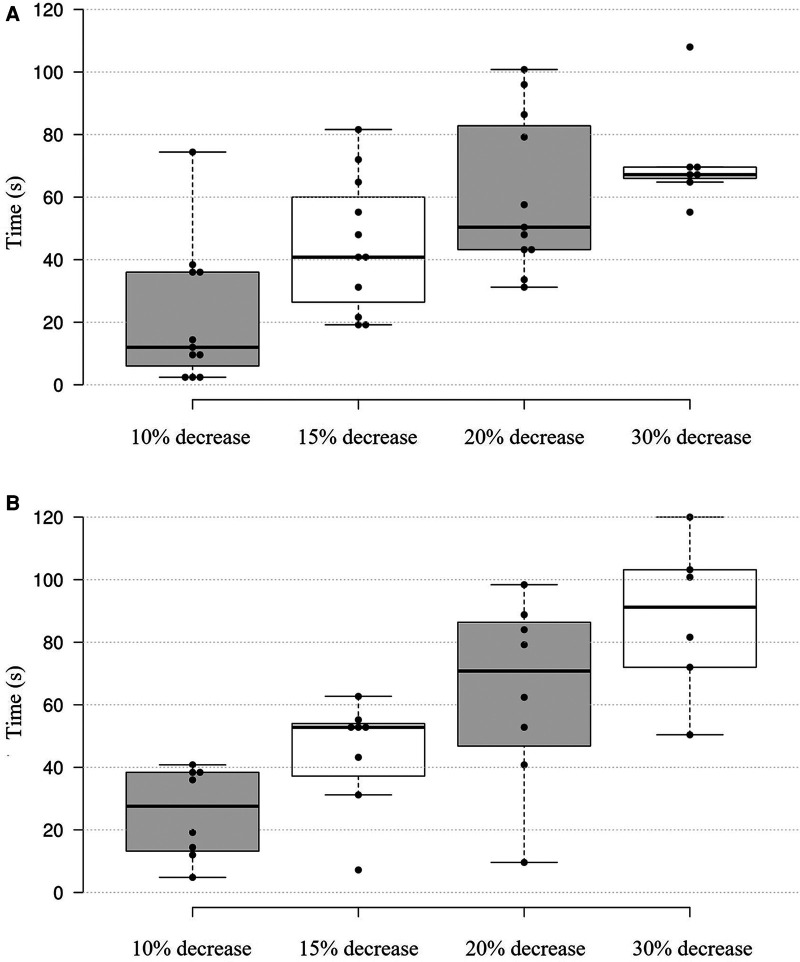
Boxplot of trunk muscles torque output, expressed as percentage of MVC5 over time: **(A)** trunk flexor muscles (during eccentric trunk extension), **(B)** trunk extensor muscles (during eccentric trunk flexion). The boxplot shows the distribution of the percentage decline in torque output in trunk flexor muscles for 11 participants and in trunk extensor muscles for 8 participants over 2-minute AO protocol. Depicted are box and interquartile ranges (75th and 25th percentiles), with median (thick line) and individual data points (dots) of each participant.

Individuals' torque output percentage decline over time is illustrated as boxplots in Figure 2. For the trunk extensor muscles, 6 out of 8 participants reached a 30% decrease in torque output, while for the trunk flexor muscles, 7 out of 11 participants reached a 30% decrease in torque output.

## 4 Discussion

The aim of the present preliminary study was to assess fatigue characteristics in an eccentric trunk flexion- extension protocol and to identify differences and the time required to induce fatigue in a population of asymptomatic individuals. Mean torque output values of the participants during the 2-minute AO protocol revealed a notable decline in torque output of up to 20% within the initial minute. Nonetheless, distinct individual variations in torque output profiles were observed among participants throughout the AO protocol. Highest interindividual variability was observed at a 10% torque output drop; however, as fatigue levels increased, the variability in torque output between individuals decreased, resulting in more homogeneous fatigue timepoints as by the defined thresholds.

Trunk muscle fatigue can occur either following sustained activity or as a consequence of repeated dynamic contractions, and its onset exhibits considerable variability influenced by factors such as the intensity, duration and type of activity (3). In general, most of the studies utilized a 30-second to 2-minute AO protocol to set off muscle fatigue (21, 26). In line with this practice, the present study employed a 2-minute AO protocol to investigate the time point at which acute fatigue is induced during maximum eccentric contractions. Moreover, fatigue studies involving maximal eccentric contractions have consistently shown a substantial decrease in muscle force, with reductions ranging from 30% to 50% following repeated contractions (27). In the current study, approximately half of the participants reached a 30% torque decrease in given time, specifically 6 out of 8 participants for trunk extensor muscles and 7 out of 11 participants for trunk flexor muscles. Regarding the average results of this study, a decrease in torque output of 20% within the first minute, and a further decrease to 30% between 90 and 120 s was observed in trunk flexor muscles. In line with this study, previous studies have reported that force decline profiles in abdominal muscles tend to exhibit slightly steeper declines compared to the back muscles, potentially due to muscle fiber composition (28–30). Compared to the trunk flexor muscles, a decrease in torque output of 15% was observed within the first one minute but a more significant decrease to 30% was observed between 60 and 120 s in the trunk extensor muscles. This suggests that inducing significant fatigue in the trunk extensor muscles may require a prolonged protocol compared to the trunk flexor muscles. Previous research has indicated that fatigue induced changes in torque output during eccentric trunk flexion tend to display a relatively consistent and gradual decline over time, in contrast to tasks involving eccentric contractions in upper or lower extremity muscles (22, 31–33), supporting the results of the current study.

Noticeable individual differences were seen in force decline profiles during the AO protocol. Some participants experienced a rapid decline in torque whereas some participants maintained their torque output for a longer period. These individual differences were apparent not only within the same muscle groups, but also across the different muscle groups such as flexors and extensors. Previous studies have revealed variations in muscle activation patterns among participants, suggesting that individual neuromuscular control strategies may contribute to the differences in force decline profiles (3). These variations could be attributed to differences in muscle fiber type composition, motor unit recruitment strategies, or biomechanics of the trunk muscles (34). For instance, individuals with a greater proportion of type I muscle fibers (which are more fatigue-resistant) may show a slower decline in torque output during the protocol than individuals with a greater proportion of type II muscle fibers (which are more fatigable) (34). Similarly, individuals who use recruitment strategies with larger number of motor units may experience a more rapid decline in torque output, as their motor units become fatigued more quickly (3, 35–37). In addition, maximum effort tests, irrespective to their modality, necessitate a high level of individual motivation to achieve peak physical performance (38). When participants are highly motivated, they're more likely to push through discomfort to achieve their goal. Conversely, in cases of low motivation, they might not feel the need to endure the discomfort. This can negatively affect how well they perform in activities that require maximum effort (38). Motivational factors could therefore potentially account for the observed variations in torque output across participants. Moreover, differences in force outcome could be attributed to participants’ varying levels of physical activity, lack of familiarity with eccentric exercises, disparities in coordination, and differing levels of physical fitness (39–42). The present study's findings also indicate that the varied training loads among participants might have influenced the results. Notably, participants with higher training loads exhibited smaller mean fluctuations in force output over the trial duration but demonstrated higher force variability. This might suggest that while a higher training load could lead to less decline in performance, it might also result in greater inconsistency (43, 44). Additionally, participants with higher training loads may have developed greater muscle endurance and strength, potentially affecting their response to the experimental intervention compared to those with lower training loads (45).

Neuromuscular fatigue exerts functional implications on motor output during fatiguing tasks, one of which is increased variability in motor output (46). Typically this phenomenon is explained by alterations in motor unit recruitment and discharge timing as fatigue progresses (46–48). Expanding upon this, the present study explored the dynamics of torque output variability during a fatiguing task and, in accordance with prior research, identified a decreasing pattern in torque output variability as fatigue levels increased. Consistent with the existing literature, this heightened variability was quantified through an increase in the coefficient of variation (CV) of torque output (3, 46–49). When comparing the variations in fatigue thresholds, it was revealed that as fatigue increased, the differences in torque output between individuals decreased. This implies that as fatigue levels increase, there is a more uniform distribution of fatigue timepoints among individuals.

A few limitations need to be taken into account for interpreting the study results. The small sample size of this pilot investigation, with only descriptive analysis necessitates careful interpretation of the results and limits their generalizability. The gender imbalance may impact applicability of the results, especially regarding potential gender differences in muscle fatiguability. Participant´s training hours, hence training status, varied across participants, though reported levels of perceived exertion after the exercise (as well as before) were comparable. Standardizing training loads among participants or including larger sample sizes to specifically investigate the impact of training variability is recommended for future studies. Also, while the purpose of employing data smoothening by a 5-repetition moving average filter was to alleviate (unphysiological) fluctuations in torque output data during the protocol, it's crucial to recognize that this approach may introduce limitations that warrant a critical evaluation. The methodology's focus on assessing the first time point at which an individual reaches the respective threshold for outcome presentation, may not fully account for subsequent variability and nuances in responses, highlighting a further investigation. Lastly, it is important to note that since this study solely involved asymptomatic healthy participants, caution should be exercised in applying the findings to clinical populations.

In conclusion, notable individual variations in torque output profiles during a fatiguing task were demonstrated, with some participants showing a rapid drop in torque output, while others sustained their torque output for longer durations. Regarding the duration of the protocol, our results suggest that significant decreases (up to 20% decrease) in torque output can occur within the initial 1-minute of eccentric trunk flexion and extension. Furthermore, the variation in fatigue thresholds occurring in less than 1-minute was greater than the variation in fatigue thresholds occurring over 1-minute, which indicates fatigue characteristics among individuals are more homogeneous in longer protocols. However, to induce more pronounced fatigue, a 2-minute AO protocol was needed.

The findings of this pilot investigations suggest that this protocol could be a valuable tool for researchers and clinicians in various fields, such as sports medicine and physical therapy. However, further investigations with higher sample size and assessment of potential factors individually affecting the force profiles are required. If proven true, the protocol might be used for identification of muscle fatigue in athletes or individuals who perform physically demanding tasks that involve repetitive trunk movements. By identifying early signs of fatigue, trainers and therapists, healthcare professionals might adjust training programs and workloads, thereby reducing the risk of injury and optimizing performance.

## Data Availability

The raw data supporting the conclusions of this article will be made available by the authors, without undue reservation.
