# Supplementary material for: A comparison of individual force decline profiles during a fatiguing eccentric trunk flexion and extension protocol: a pilot study
Source: Front Sports Act Living. 2024 Aug 21;6:1431607. doi: 10.3389/fspor.2024.1431607 (PMC11371616; doi:10.3389/fspor.2024.1431607)
Supplement: Supplementary file 1 [file Datasheet1.pdf]

SUPPLEMENTAL FILE

A

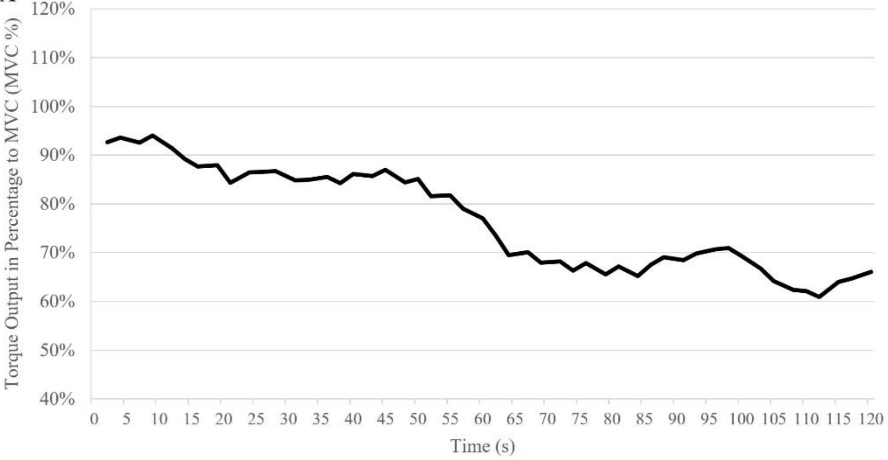

B

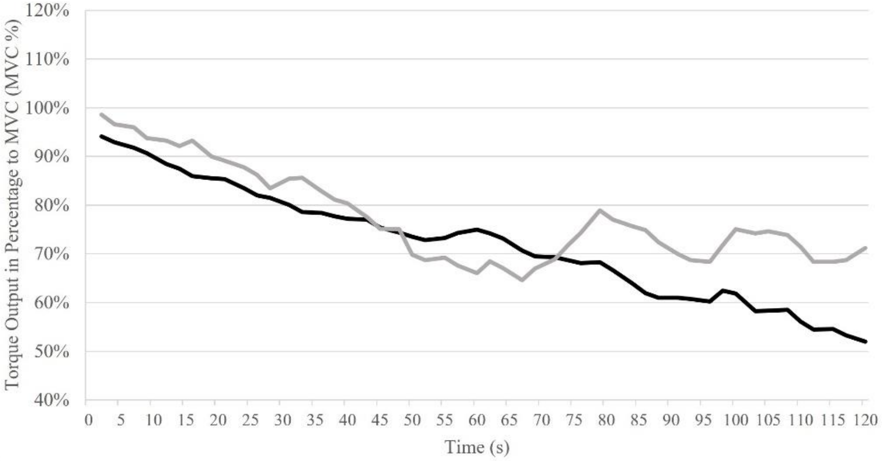

C

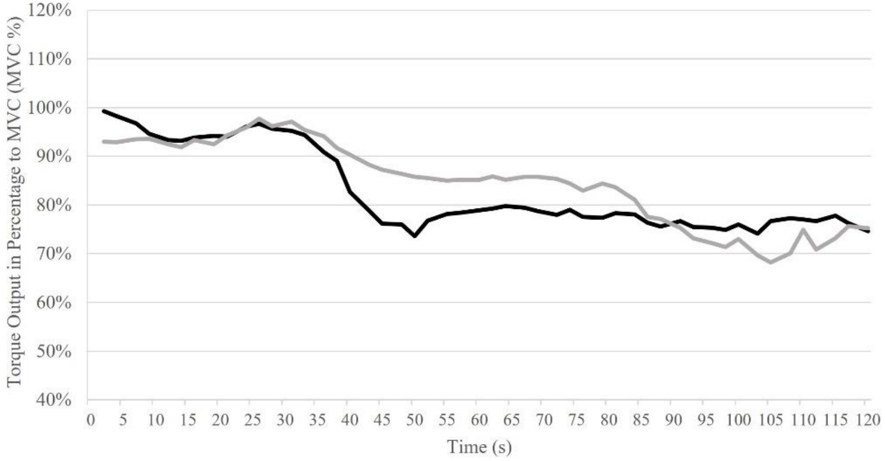

D

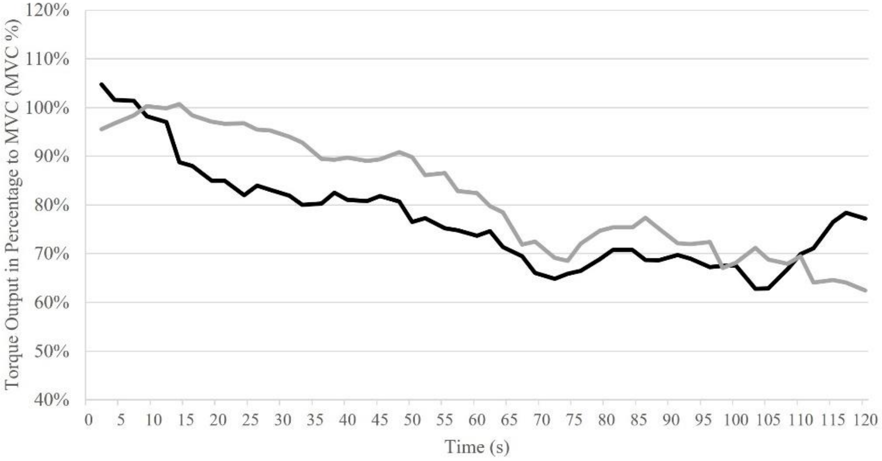

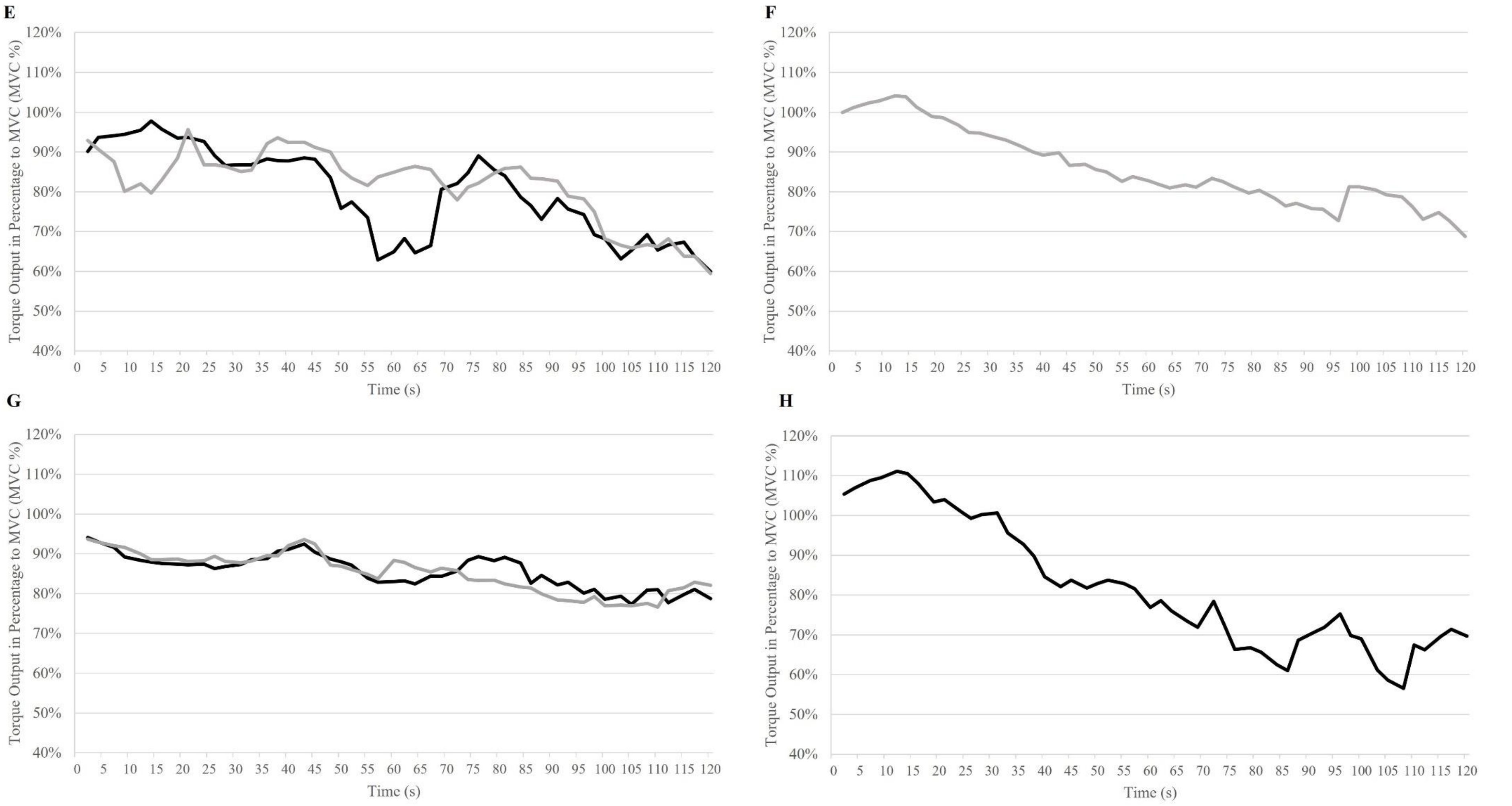
E
F
G
H
Torque Output in Percentage to MVC (MVC %)
120%
110%
100%
90%
80%
70%
60%
50%
40%
0 5 10 15 20 25 30 35 40 45 50 55 60 65 70 75 80 85 90 95 100 105 110 115 120
Time (s)

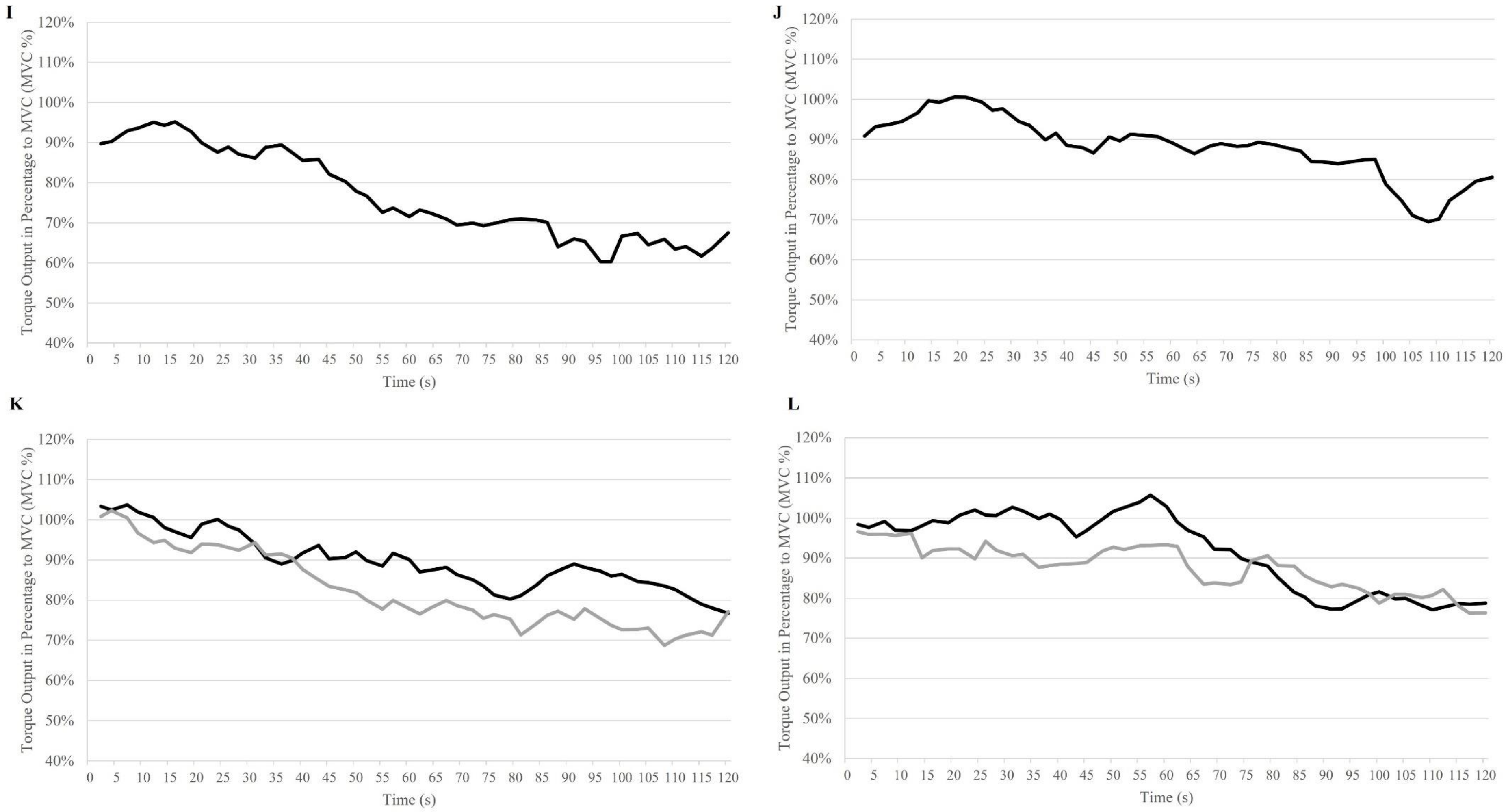

Trunk flexion/extension torque output during the 2-minute AO task (Individual torque decline profiles during a fatiguing eccentric trunk flexion and extension protocol. The graph shows the percentage of maximal voluntary contraction (MVC%) over time for 12 individuals (A-L). Black lines represent trunk flexor muscle torque, and grey lines represent trunk extensor muscle torque.
